# 

**Published:** 1878-08

**Authors:** 


PAMPHLETS RECEIVED.—Vascular Tumors of the Female Urethra: with the description of a speculum devised to facilitate their removal. By A. Reeves Jackson, M.D., Chicago, Ill.

The Application of Pressure in Diseases of the Uterus. By V. H. Taliaferro, Professor of Obstetrics and Diseases of Women and Children in the Atlanta Medical College.

Yellow Fever, the Epidemic of 1876 in Savannah. By J. C. LeHardy, M.D. Savannah, Ga. Reprint from the Transactions of the Medical Association of Georgia.

Sterility and its Treatment. By William H. Walthen, M.D., Clinical Lecturer on Diseases of Women and Children, Louisville; Medical Surgeon to the Female Department Louisville City Hospital. Reprint from the Transactions of the Kentucky State Medical Society. 1877. Louisville, Ky.

Certain Symptoms of Nervous Exhaustion. By George M. Beard, M.D., etc. Read before the New York Academy of Medicine April 4, 1878. Reprint from Virginia Medical Monthly.

Remarks on Social Conservatism. By J. W. Singleton, M.D., Paducah, Ky. Read before the Medical Society of Illinois, Indiana and Kentucky, at Evansville, Ind. 1877.

Cholecystotomy for the Removal of Gall-stones in Dropsy of the Gall-bladder. By J. Marion Sims, M.D., founder of the Woman's Hospital, New York, and formerly Surgeon of the same, etc., etc. Reprint from the *British Medical Journal*, June 8, 1878, London.

Minutes of the State Medical Society of Arkansas, at its Third Annual Session, Little Rock. 1878.

A New Rotating Urethrotome. By John A. Pritchett, Hayneville, Ala. Reprint from the *New York Medical Journal*, July, 1878.

The Soft Palate—its Value in Diagnosis. By Wm. Abram Love, M.D., Professor of Physiology in Atlanta Medical College.

---

College Announcements Received.—Atlanta Medical College opens on 15th October and closes March 4th.

Savannah Medical College, November 4th, March 4th.

Medical College of Evansville, Indiana, October 1st, March 1st.

University of Pennsylvania, October 1st, March 1st.

Bellevue Hospital Medical College, October 2d.

Medical College of South Carolina, October 15, early in March.

College of Physicians and Surgeons, New York, October 1st, March 1st.

Louisville Medical College, October 1st, March 1st.

Medical College of Georgia, Augusta, Ga., October 1st, March 1st.

University of Georgetown, D. C., September 2d, April.

As the busy season is fast approaching, we must remind those of our subscribers who are in arrears that we are prepared to receive their remittances. Many of them have written us during the summer that they would pay in the fall. We trust we shall hear from them soon.

